# On-Surface Synthesis of Anthracene-Fused Zigzag Graphene Nanoribbons from 2,7-Dibromo-9,9′-bianthryl Reveals Unexpected Ring Rearrangements

**DOI:** 10.1021/prechem.3c00116

**Published:** 2024-02-11

**Authors:** Xiushang Xu, Amogh Kinikar, Marco Di Giovannantonio, Carlo A. Pignedoli, Pascal Ruffieux, Klaus Müllen, Roman Fasel, Akimitsu Narita

**Affiliations:** † Max Planck Institute for Polymer Research, 55128 Mainz, Germany; ‡ Empa, Swiss Federal Laboratories for Materials Science and Technology, nanotech@surfaces Laboratory, 8600 Dübendorf, Switzerland; § Organic and Carbon Nanomaterials Unit, Okinawa Institute of Science and Technology Graduate University, 1919-1 Tancha, Onna-son, Kunigami-gun, Okinawa 904-0495, Japan; ∥ Institute of Physical Chemistry, Johannes Gutenberg University Mainz, Duesbergweg 10-14, 55128 Mainz, Germany; ⊥ Department of Chemistry, Biochemistry and Pharmaceutical Sciences, University of Bern, Freiestrasse 3, 3012 Bern, Switzerland; # Institute of Structure of Matter − CNR (ISM-CNR), via Fosso del Cavaliere 100, 00133 Roma, Italy

**Keywords:** On-surface synthesis, Graphene nanoribbon, On-surface reaction, Rearrangement, Edge state

## Abstract

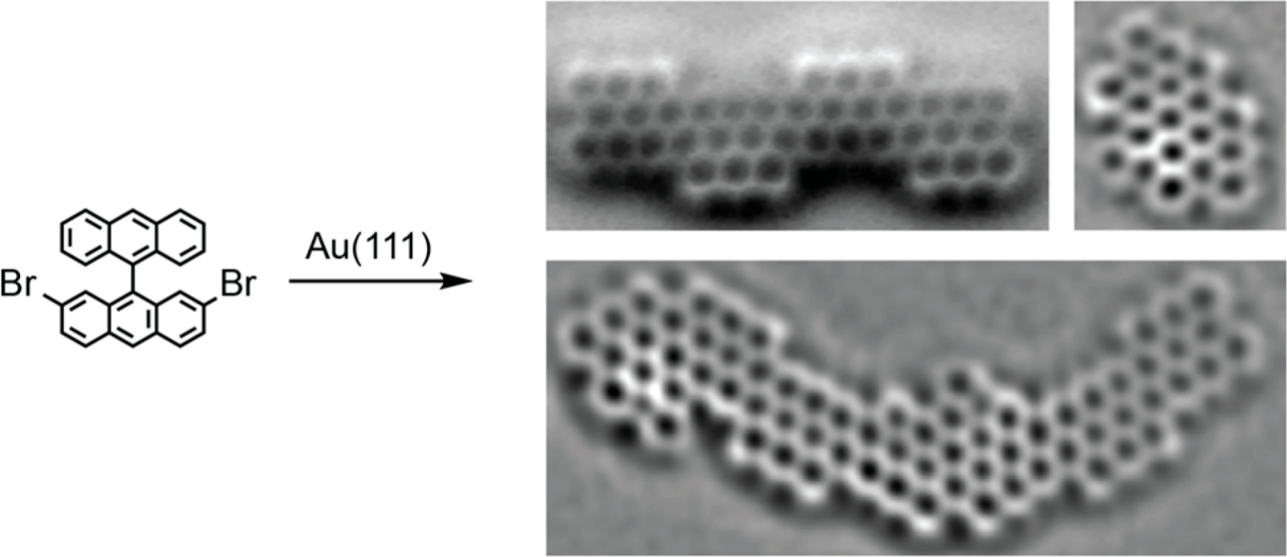

On-surface synthesis has emerged as a powerful strategy to fabricate unprecedented forms of atomically precise graphene nanoribbons (GNRs). However, the on-surface synthesis of zigzag GNRs (ZGNR) has met with only limited success. Herein, we report the synthesis and on-surface reactions of 2,7-dibromo-9,9′-bianthryl as the precursor toward π-extended ZGNRs. Characterization by scanning tunneling microscopy and high-resolution noncontact atomic force microscopy clearly demonstrated the formation of anthracene-fused ZGNRs. Unique skeletal rearrangements were also observed, which could be explained by intramolecular Diels–Alder cycloaddition. Theoretical calculations of the electronic properties of the anthracene-fused ZGNRs revealed spin-polarized edge-states and a narrow bandgap of 0.20 eV.

## Introduction

**Figure 1:**
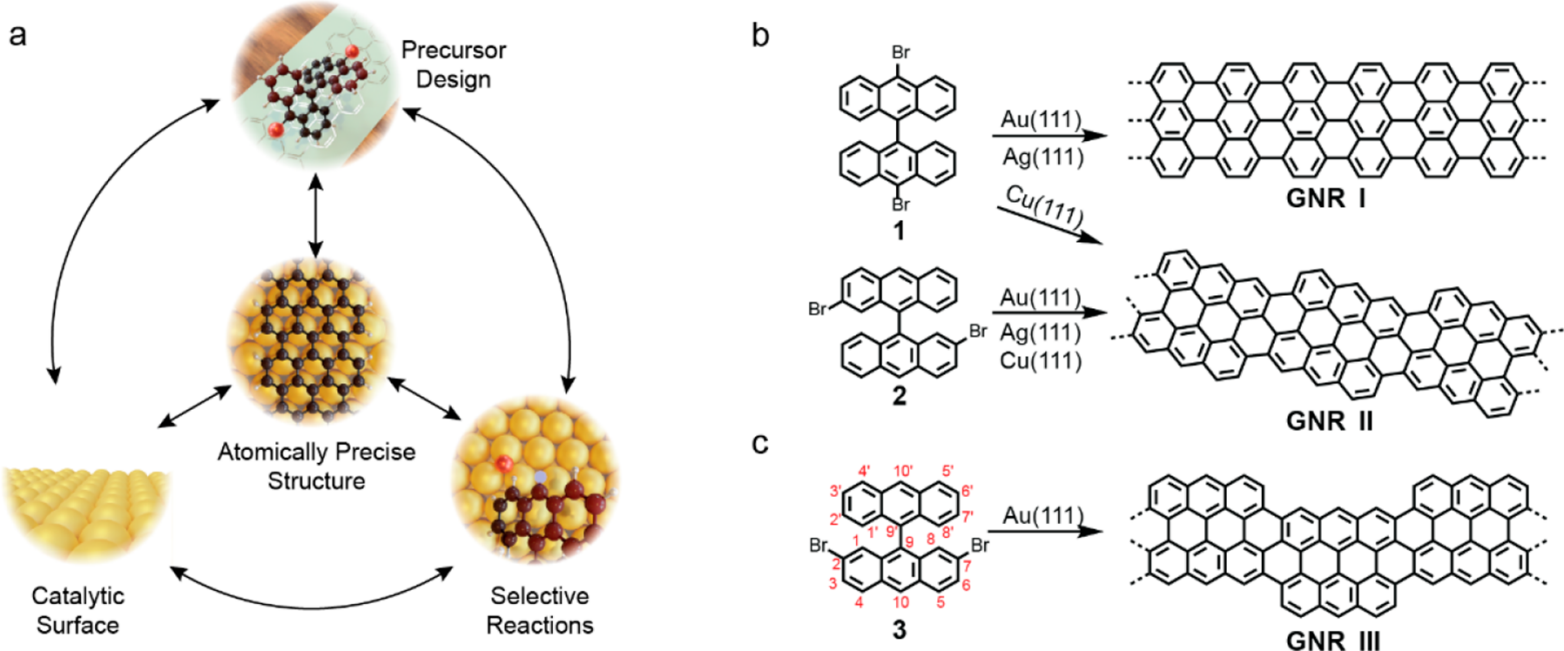
(a) Illustration of the fundamental concepts in the on-surface synthesis of atomically precise GNRs. (b) The chemical structure of 7-AGNR (GNR I) and (3,1)-GNR (GNR II) obtained from 10,10′-dibromo-9,9′-bianthryl (**1**) and 2,2′-dibromo-9,9′-bianthryl (**2**), respectively. (c) The structure of 3-ZGNR-EA (GNR III) using 2,7-dibromo-9,9′-bianthryl (**3**) as the precursor, introduced in this work.

Graphene nanoribbons (GNRs) have been attracting increasing interest in view of their unique electronic and magnetic properties and potential applications in nanoelectronics and quantum information technologies.^1−3^ The electronic properties of GNRs, including bandgap, charge-carrier mobility, and degree of spin-polarization, depend sensitively on their chemical structure.^4−6^ This makes it important to obtain GNRs with atomic precision, which can be achieved by bottom-up molecular synthesis.^2,4,6,7^ On-surface synthesis under ultrahigh vacuum (UHV) conditions has emerged as a powerful method that is complementary to conventional solution synthesis. It realizes atomically precise GNRs on metal surfaces and allows for their in situ atomically resolved visualization and spectroscopic characterization by scanning probe methods.^7,8^ The on-surface synthesis of GNRs relies on 1) the design of molecular precursors leading to the targeted GNR structures, 2) the use of catalytic metal surfaces (usually Au, Ag and Cu single crystals), and 3) the achievement of selective on-surface reactions (typically dehalogenative homocoupling^9^ and cyclodehydrogenation^10^), which are all interlinked (Figure 1a).^4,11^ Importantly, precise chemical structures of individual products can be directly visualized on the surface, sometimes revealing surface-catalyzed reactions that have no equivalent in solution chemistry and provide essential feedback for the precursor design.

Since the initial report in 2010 on the on-surface synthesis of 7-atom-wide armchair GNRs (7-AGNRs) using 10,10′-dibromo-9,9′-bianthryl (10,10′-DBBA **1**) as the molecular precursor (Figure 1a),^12^ a great number of GNRs with different edge structures have been developed. For example, AGNRs with varying widths,^13−19^ chiral GNRs,^20,21^ chevron-type GNRs,^12,22^ and heteroatom-doped GNRs^23−25^ have been achieved. In particular, on-surface reactions of monomer **1** using Cu(111) as the metal substrate afforded chiral (3,1)-GNR,^20,26^ which was later also obtained on Au(111) and Ag(111) using 2,2′-dibromo-9,9′-bianthryl (2,2′-DBBA **2**) as the monomer (Figure 1b).^27^ Multiple functionalized derivatives of 10,10′-DBBA **1** have also been explored, enabling π-extension of 7-AGNRs^28,14^ and further fusion into nanoporous graphene.^29^ Moreover, lateral extension of chiral (3,1)-GNR was recently demonstrated through substitution of 2,2′-DBBA **2** with two additional anthryl groups. 10,10′-DBBA **1** and 2,2′-DBBA **2** with different positions of the bromo substitutions thus led to the synthesis of distinct GNRs, allowing also for further π-extension. However, other pristine DBBA isomers have not been synthesized and investigated on surfaces to date.

Zigzag GNRs (ZGNRs) can host spin-polarized states localized on their edges and are thus considered promising for spintronic device applications.^3,30,31^ However, experimental reports on ZGNRs are still scarce in literature.^32−36^ To this end, we have recently proposed a new design motif for the synthesis of edge-extended ZGNRs, obtaining bisanthene-fused ZGNRs.^33^ Based on this motif, 2,7-dibromo-9,9′-bianthryl (2,7-DBBA **3**) can be considered to furnish anthracene-fused *N* = 3 ZGNRs, which we name as 3-ZGNR-E(Anthracene, 7) (see ref (33). for the definition of the nomenclature, where E stands for “Extended” and 7 indicates the periodicity of the fused units in terms of the axial unit vector of the ZGNR; hereafter called 3-ZGNR-EA for clarity). Here we report the solution synthesis of 2,7-DBBA **3** and its on-surface reactions toward 3-ZGNR-EA. The reaction products on Au(111) were unambiguously characterized by scanning tunneling microscopy (STM) and noncontact atomic force microscopy (nc-AFM), revealing the successful formation of 3-ZGNR-EA (up to 7 repeating units) as well as unanticipated isomerization with concomitant ring rearrangement. Moreover, the electronic properties of 3-ZGNR-EA were studied by density functional theory (DFT) calculations, elucidating its small bandgap as well as its spin-polarized frontier states.

## Result and Discussion

**Scheme 1:**
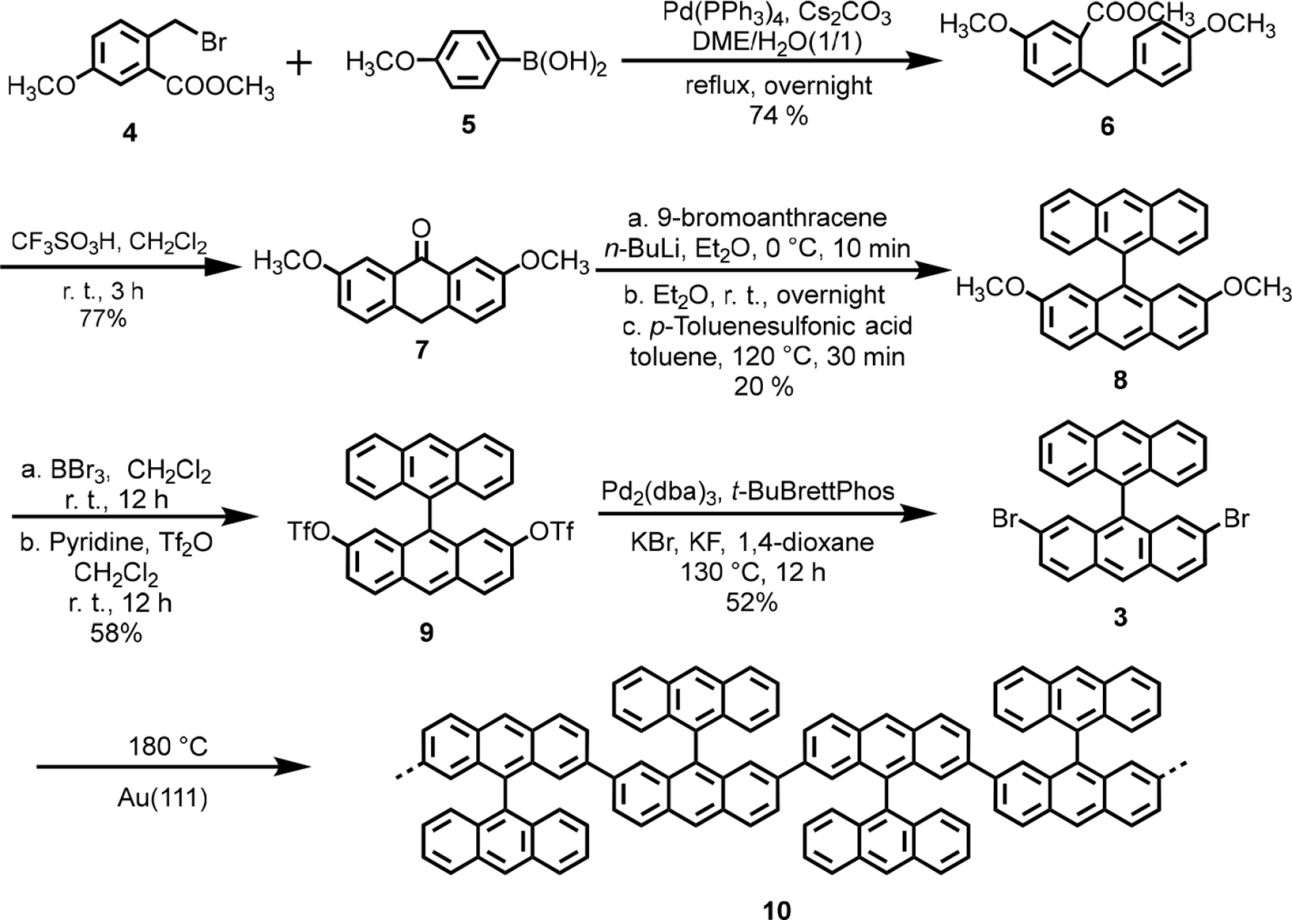
Synthetic Route to 2,7-Dibromo-9,9′-bianthryl (**3**) DME: 1,2-dimethoxyethane.

The synthetic route to 2,7-DBBA **3** is described in Scheme 1. Methyl 5-methoxy-2-(4-methoxybenzyl)benzoate (**6**) was obtained by Suzuki-Miyaura coupling of 2-(bromomethyl)-5-methoxybenzoate (**4**) and (4-methoxyphenyl)boronic acid (**5**) in 74% yield. Subsequently, **6** was cyclized by trifluoromethanesulfonic acid to afford 2,7-dimethoxyanthracen-9(10*H*)-one (**7**) in 77% yield. Then, 9-bromoanthrancene was lithiated to anthracen-9-yllithium, and subsequently reacted with **7**, followed by dehydroxylation using a catalytic amount of *p*-toluenesulfonic acid to produce 2,7-dimethoxy-9,9′-bianthryl (**8**) in 20% yield. After demethylation of the methoxy groups of **8** with boron tribromide (BBr_3_), the resulting diol was reacted with trifluoromethanesulfonic (Tf_2_O) to afford bistriflate **9** in 58% yield. Finally, 2,7-DBBA **3** was obtained by palladium (Pd)-catalyzed conversion of **9** in 52% yield,^37^ and characterized by ^1^H and ^13^C NMR spectroscopies and high-resolution mass spectrometry (see the Supporting Information).

**Figure 2:**
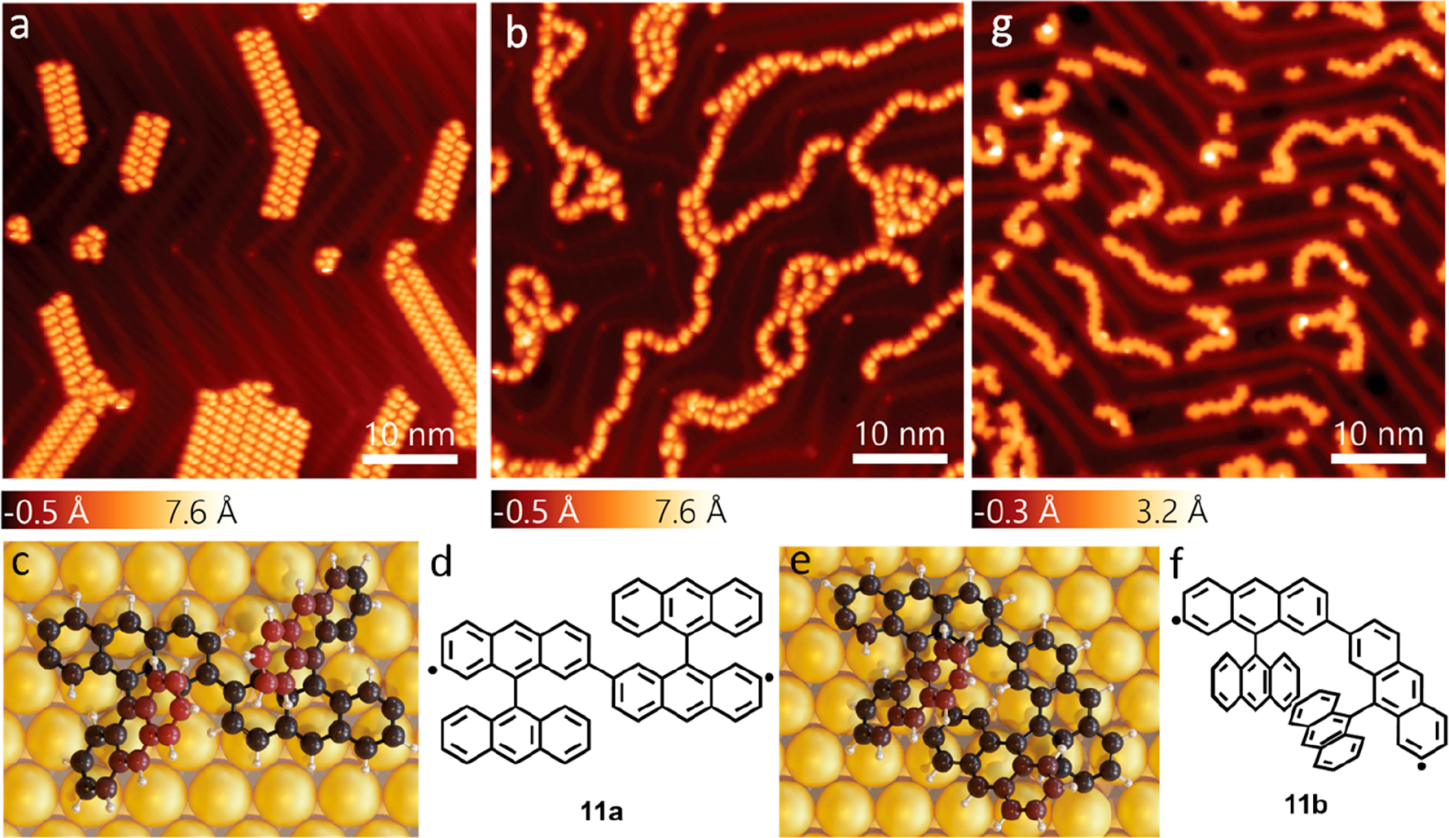
(a) STM image acquired after depositing **3** on Au(111) at room temperature (tunneling parameters: *V* = 1 V, *I* = 10 pA). (b) STM image acquired after annealing the sample to 180 °C, showing the formation of long polymer chains (*V* = 1 V, *I* = 20 pA). (c, e) DFT optimized geometries of the chemical structures indicated on the right side (d, f). (see the Supporting Information for details about the calculations). The carbon atoms further away from the surface plane are colored brown to emphasize the nonplanar structure. (g) STM image acquired after annealing to 355 °C, showing the cyclodehydrogenation of the polymers to planar GNRs (*V* = 50 mV, *I* = 100 pA). (Color bars for a, b, and g indicate the apparent height).

Toward the on-surface synthesis of 3-ZGNR-EA, 2,7-DBBA **3** was deposited on a clean Au(111) surface by sublimation under UHV conditions. An STM image of the resulting Au(111) surface revealed individual molecules self-assembled into rows that were closely packed into islands (Figure 2a). Long polymer chains were observed after annealing at 180 °C, indicating formation of polymer **10** through the cleavage of the C–Br bonds of **3** to generate diradical intermediates, followed by aryl–aryl coupling (Scheme 1 and Figure 2b). The formation of long polymers could be validated by tip manipulation (see Figure S1 in the Supporting Information). This result is in contrast to the results obtained with some previous dihalogenated precursors.^38^ These failed to polymerize on surface, and reflected the fact that the radical sites of the intermediates from **3** are sterically accessible, presumably due to favorable twisting angles of the anthryl groups pointing out of the plane of the surface. Nevertheless, the observed polymer chains displayed irregular kinks (Figure 2b), which could be explained by the presence of different conformers. Taking the dimeric intermediate as an example, two conformers **11a** and **11b** can be considered, which respectively lead to a straight polymer segment and a kink. DFT calculations revealed that **11b** is more stable on the Au(111) surface than **11a** by approximately 0.38 eV, in line with the observation of multiple kinks in polymer **10** (the optimized geometries of the molecules on the Au-slab are shown in Figure 2d,f).

**Figure 3:**
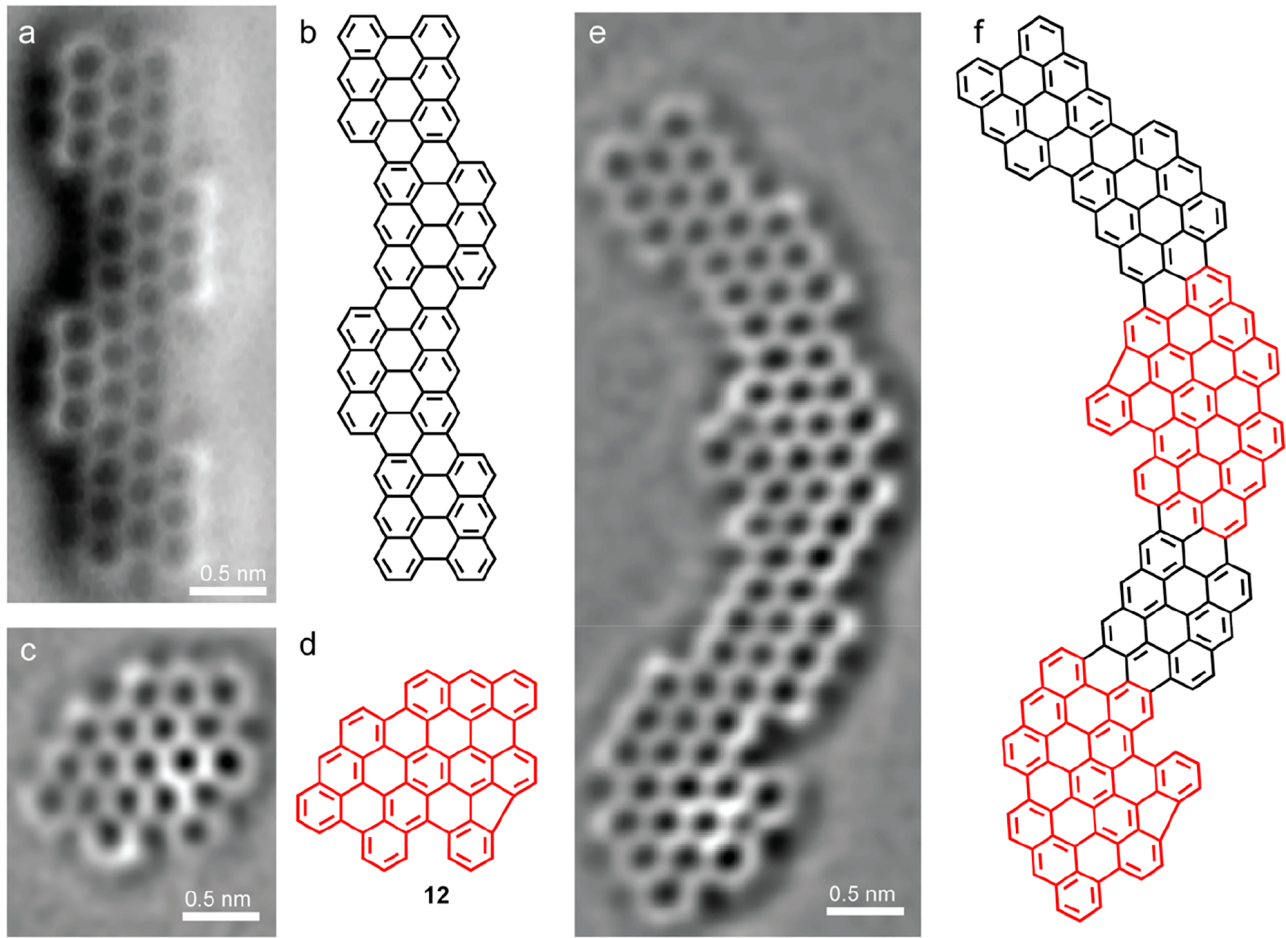
(a) Bond-resolved nc-AFM image acquired over a 4-unit long 3-ZGNR-EA with its assigned chemical structure shown in b. (c) nc-AFM image of a planarized dimer **12**, corresponding to **11b**. This image has been Laplace filtered to resolve the formation of the 5-membered ring. The assigned chemical structure is shown in d. (e) Laplace filtered nc-AFM image of a GNR segment formed with bent segments interspersed with segments of 3-ZGNR-EA. The assigned chemical structure is shown in f. See Figure S2 in the Supporting Information for additional STM and unfiltered nc-AFM images.

After further annealing to ∼350 °C, polymer **10** underwent cyclodehydrogenation to yield planar GNRs (Figure 2g). The obtained GNRs displayed worm-like structures, consisting of mixtures of straight and bent segments, which apparently originated from different conformations of polymer **10** corresponding to **11a** and **11b**, respectively. The occasional bright spots seen on the GNR segments (Figure 2g) can be attributed to structures that are not fully planarized (see the discussion of possible reaction pathway below). To identify the chemical structure of the obtained GNRs, we employed nc-AFM imaging using a CO-functionalized tip.^39^ As shown in Figure 3a, a straight GNR segment consisting of four precursor units was revealed to be the targeted 3-ZGNR-EA (Figure 3b, see also Figure S2 in the Supporting Information). On the other hand, an nc-AFM image of a bent dimer (Figure 3c) allowed us to identify its structure (**12**) as shown in Figure 3d, indicating the occurrence of a rearrangement during the cyclodehydrogenation reaction. The same structure could be identified for the bent segments of longer GNRs interspersed with the straight segments (up to 7-units long, see Figure S3 in the Supporting Information) of 3-ZGNR-EA (Figure 3e,f).

**Figure 4:**
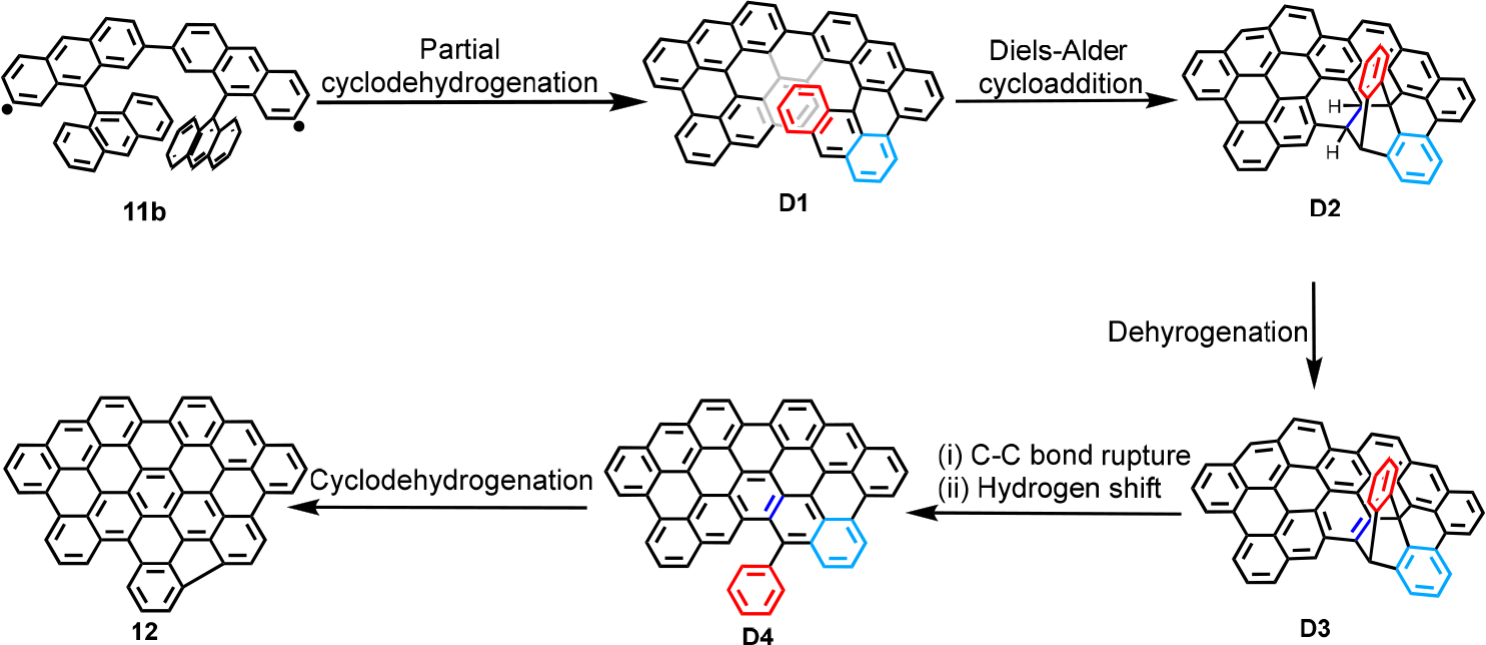
Possible reaction pathway for the formation of **12**.

A possible reaction pathway for the formation of **12** on Au(111) is proposed as shown in Figure 4. The rearrangement reaction is presumably initiated after partial cyclodehydrogenation of **11b**, enhancing the strain on the molecule during the planarization on surface.^40^ The radical sites of **11b** are most probably quenched by hydrogen atoms released during the partial cyclodehydrogenation. Here we assume intermediate **D1** with a [7]helicene substructure, forming four C–C bonds from **11b**, although other intermediate structures with less C–C bonds can also be considered (see Figure S5). According to previous discussions in the literature,^41−43^ on-surface rearrangements of nonplanar helical structures, including π-extended [7]helicene, are considered to proceed through intramolecular Diels–Alder cycloaddition. This hypothesis could also be applicable in the current case: the intramolecular Diels–Alder cycloaddition of **D1** affords intermediate **D2**. The subsequent dehydrogenation results in intermediate **D3**, which may undergo a C–C bond rupture along with a hydrogen shift to give intermediate **D4**, similar to a previous observation by Starý et al.^41^ Further cyclodehydrogenation of intermediate **D4** can then provide **12**. The bright protrusions of GNR segments ascribed to partially planarized structures in Figure 2g may correspond to intermediates **D1**–**D3**. Occasionally, GNR segments corresponding to **D4** without phenyl group were observed (see Figure S4 in the Supporting Information), which can be ascribed to the extrusion of benzyne.^41^ These results provide a rare insight into on-surface rearrangement reactions,^41−43^ which have far less been explored compared to other types of on-surface reactions, such as dehalogenative coupling, direct C–H activation,^44,45^ dehydro-Diels–Alder,^46^ and intramolecular cyclodehydrogenation reactions.^7,47,48^

**Figure 5:**
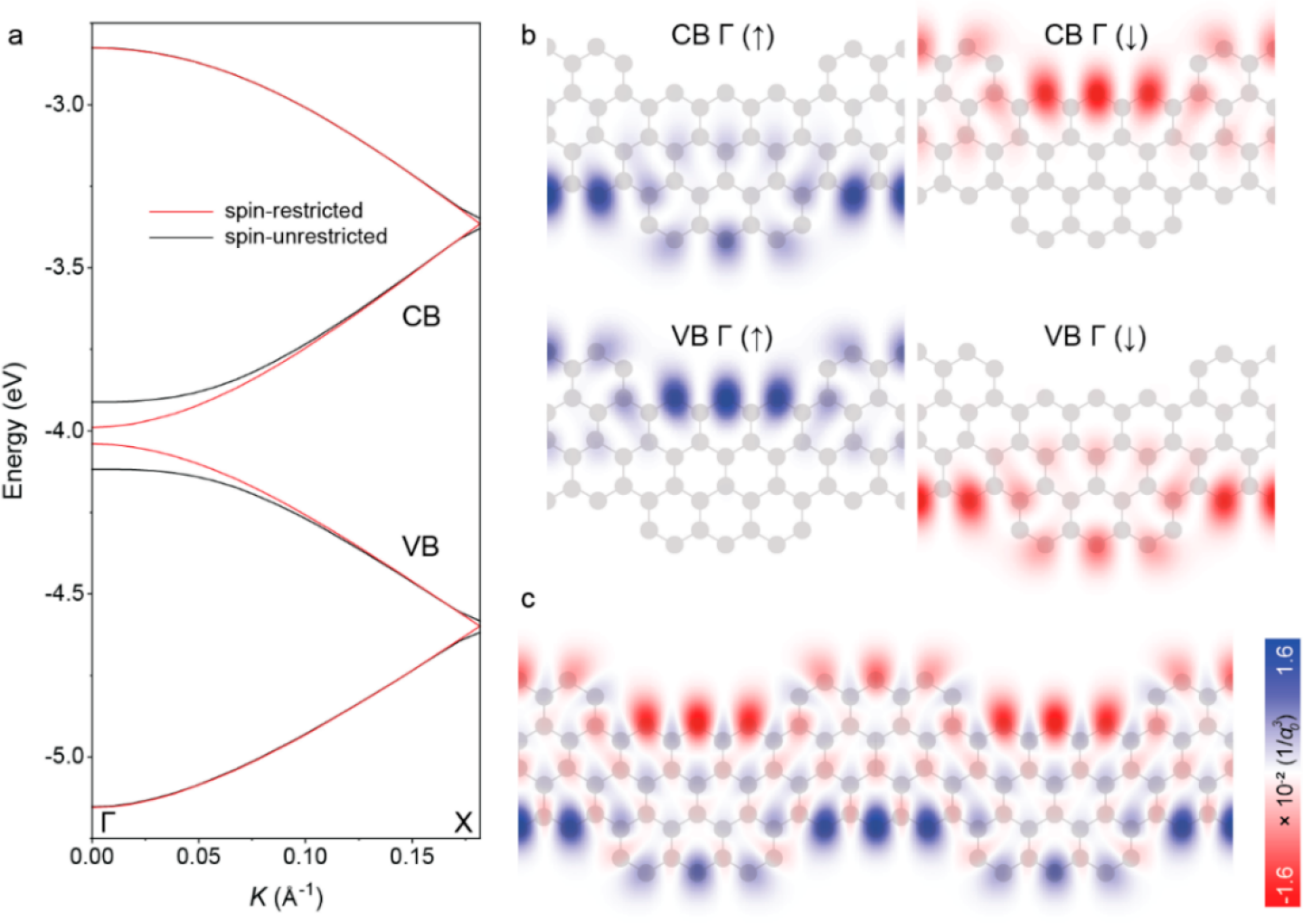
(a) Spin-restricted and spin-unrestricted DFT band-structures for 3-ZGNR-EA in gas-phase. (b) The spin-up (↑) and spin-down (↓) orbitals at the Γ point for CB and VB exhibit spin-polarization for the spin-unrestricted calculations. (c) Spin density map of 3-ZGNR-EA showing the spin-polarized edge-states. (Color bar shows the spin-density per cubic Bohr radius (*a*_,0_^3^).)

To elucidate the electronic properties of 3-ZGNR-EA, we performed spin-restricted and spin-unrestricted DFT calculations. These calculations show that upon including the spin-degree of freedom (i.e., in the spin-unrestricted case) the bandgap of the system increases from 50 to 200 meV (Figure 5a) as a result of electron–electron interactions. Moreover, the electron-density maps for the wave functions at the Γ-point for the conduction band (CB) and for the valence band (VB) are clearly spin-polarized, with the spins localizing on opposite edges of the GNR (Figure 5b), evidencing the spin-polarized edge-states (Figure 5c). Additionally, the spin-unrestricted case is more stable by 13 meV, indicating an open-shell ground state of 3-ZGNR-EA. Crucially, these states are at the same time the extremal states for the respective bands and are thus the frontier states of the 3-ZGNR-EA. This is in contrast with the pristine ZGNRs, where the maximally spin-polarized states lie deeper in the bands at the Χ-point.^49^ However, the frontier states, i.e. CB minimum or VB maximum, would dominate the electron transport in devices.^50^ In the case of 3-ZGNR-EA, as opposed to the pristine ZGNRs, these states would be the maximally spin-polarized states.

In conclusion, we have synthesized 2,7-dibromo-9,9′-bianthryl as a precursor for synthesizing an edge-extended ZGNR and explored their thermally induced on-surface reactions on the Au(111) surface. Detailed structural characterization of the products was performed by high-resolution nc-AFM and STM, revealing the formation of the anthracene-fused ZGNR (3-ZGNR-EA) segments along with unexpected, structurally rearranged segments. The rearrangement reaction can be explained by an intramolecular Diels–Alder cycloaddition during the planarization by the cyclodehydrogenation, and potentially utilized in the design of future precursors for synthesizing unprecedented structures on surface. Moreover, the electronic properties of 3-ZGNR-EA were studied by DFT calculations, elucidating intriguing spin-polarized edge-states which make 3-ZGNR-EA technologically relevant as channel materials in spintronic devices. Our results also offer valuable insights into the use of the 2,7-dibromoanthracene-based coupling motif and demonstrate the potential for synthesizing a wider variety of π-extended ZGNRs with nontrivial electronic states.

## Supporting Information Available

Synthetic details, additional STM and nc-AFM measurements results, and NMR spectra (PDF)

## Notes

The data that support the findings of this study are available in Materials Cloud Archive at http://doi.org/10.24435/materialscloud:fq-nq.
