# Supplementary material for: On-Surface Synthesis of Anthracene-Fused Zigzag Graphene Nanoribbons from 2,7-Dibromo-9,9′-bianthryl Reveals Unexpected Ring Rearrangements
Source: Precis Chem. 2024 Feb 11;2(2):81–7. doi: 10.1021/prechem.3c00116 (PMC10900509; doi:10.1021/prechem.3c00116)
Supplement: Supplementary file 1 — pc3c00116_si_001.pdf [file pc3c00116_si_001.pdf]

Supporting Information On-Surface Synthesis of Anthracene-Fused Zigzag Graphene Nanoribbons from 2,7-Dibromo-9,9'-bianthryl Reveals Unexpected Ring Rearrangements

Xiushang Xu,[#, a, c] Amogh Kinikar,[#, b] Marco Di Giovannantonio,[b,f] Carlo A. Pignedoli,[a] Pascal Ruffieux,[b] Klaus Müllen,*[,a, d] Roman Fasel,*[,b,e] Akimitsu Narita*[,a, c]

[a] Max Planck Institute for Polymer Research, 55128 Mainz, Germany.

[b] Empa, Swiss Federal Laboratories for Materials Science and Technology, nanotech@surfaces Laboratory, 8600 Dübendorf, Switzerland

[c] Organic and Carbon Nanomaterials Unit, Okinawa Institute of Science and Technology Graduate University, 1919-1 Tancha, Onna-son, Kunigami-gun, Okinawa 904-0495, Japan

[d] Institute of Physical Chemistry, Johannes Gutenberg University Mainz, Duesbergweg 10-14, 55128 Mainz, Germany

[e] University of Bern, Department of Chemistry, Biochemistry and Pharmaceutical Sciences, Freiestrasse 3, 3012 Bern, Switzerland

[f] Institute of Structure of Matter – CNR (ISM-CNR), via Fosso del Cavaliere 100, 00133 Roma

[#]These authors contributed equally to this work.

*Corresponding authors: Klaus Müllen, E-mail： muellen@mpip-mainz.mpg.de; Roman Fasel, E-mail: roman.fasel@empa.ch; Akimitsu Narita, E-mail: akimitsu.narita@oist.jp.

General Methods

1. Synthesis and characterizations

All reactions working with air- or moisture-sensitive compounds were carried out under an argon atmosphere using standard Schlenk line techniques. Unless otherwise noted, all starting materials and other chemicals were purchased from commercial sources and used without further purification. Thin layer chromatography (TLC) was done on silica gel coated aluminum sheets with F254 indicator and column chromatography separation was performed with silica gel (particle size 0.063-0.200 mm). Nuclear Magnetic Resonance (NMR) spectra were recorded using Bruker DPX 300 and Bruker DPX 400 MHz NMR spectrometers. Chemical shifts (δ) were expressed in ppm relative to the residual solvents (DMSO-d_6: ^{1}H: 2.50 ppm, ^{13}C: 39.53 ppm; CD_2Cl_2, ^{1}H: 5.32 ppm, ^{13}C: 53.84 ppm; $CDCl_3$, ^{1}H: 7.26 ppm, ^{13}C: 76.00 ppm). Coupling constants (J) were recorded in Hertz. High-resolution mass spectra (HRMS) were recorded on a Bruker Reflex II-TOF spectrometer by matrix-assisted laser decomposition/ionization (MALDI) using 7,7,8,8-tetracyanoquinodimethane (TCNQ) as matrix and calibrating with poly(ethylene glycol).

2. Sample preparation

Ultrahigh vacuum (UHV) experiments were performed on single crystal Au(111) substrates (MaTeck GmbH). The substrates were cleaned by repeated cycles of sputtering with Ar^+-ions and subsequent annealing to 400-450 °C. A custom built evaporator was used to deposit the precursors onto the clean substrate by sublimation. The sublimation temperature was adjusted to obtain a deposition rate of ~0.04 monolayer /min. The substrates were annealed to the specified temperature for 15-60 min to trigger the on-surface reactions. Sample temperatures were measured with a pyrometer (Optris). All sample preparations were performed in a preparation chamber (base pressure: 2×10^{-10} mbar) and transferred to the attached STM chamber (base-pressure: 7×10^{-11} mbar) for analysis.

3. Scanning tunneling microscopy (STM) and non-contact atomic force microscopy (nc-AFM) measurements

The STM and nc-AFM experiments were performed using a low-temperature scanning tunneling microscope (Scienta Omicron) operated at 4.7 K with a tungsten tip placed on a qPlus® tuning fork sensor.[1] The tip was functionalized with a single CO molecule at the tip apex picked up from the previously CO-dosed surface.[2] The CO molecule was picked up from the Au(111) surface by scanning the surface at –20 mV and 120-200 pA. The sensor was driven at its resonance frequency (22255 Hz, quality factor 30.8k) with a constant amplitude < 100 pm. The nc-AFM images shown in the main text have been Laplace filtered to enhance the contrast. The raw data is shown in the supporting information. The frequency shift from the resonance of the tuning fork was recorded in constant-height mode using Omicron Matrix electronics and HF2Li PLL by Zurich Instruments. The Δz is positive when the tip-

surface distance is increased with respect to the STM set point and negative when the tip-surface distance is decreased. Δz is set to zero when the feedback loop is switched off.

4. Computational details

The density functional theory (DFT) calculations were executed using the AiiDAlab platform[3] based on AiiDA[4] and the CP2K code.[5] To emulate the surface-adsorbate interaction, a repeated slab method was adopted. The simulation cell consisted of 4 atomic layers of Au along the [111] direction. One side of the slab was passivated by a layer of hydrogen atoms to suppress the Au(111) surface state. To decouple the simulation cell from its periodic replicas 40 Å of vacuum was included in the simulation cell in the direction perpendicular to the surface. Electronic states were expanded using a TZV2P Gaussian basis set[6] for C and H species and a DZVP basis set for Au species. For the plane-wave basis set, a cutoff of 600 Ry was utilized. The core electrons of the atoms were represented using norm-conserving Goedecker-Teter-Hutter pseudopotentials.[7] The exchange-correlation functional's generalized gradient approximation[8] was approached via the PBE parameterization, while van der Waals interactions were treated using Grimme's D3 scheme.[9] The gold surface was modeled using a supercell of size 29.48 × 30.64 Å^2 (equivalent to 600 Au atoms). Equilibrium geometries were ascertained by fixing the bottom two slab layers to their ideal bulk positions and relaxing the remaining atoms until forces dropped below 0.005 eV/Å. For nc-AFM simulations in AiiDAlab, the equilibrium geometries and CP2K-derived electrostatic potential were used with Hapala's probe particle code.[10] The GNR's DFT band structure calculations were performed with the Quantum Espresso software package[11] and employed the PBE exchange-correlation functional. A plane wave basis with an energy cutoff of 400 Ry for the charge density was used together with PAW pseudopotentials (SSSP)[12] and a Monkhorst k-mesh of 13 × 1 × 1. The cell and atomic geometries were relaxed until forces were smaller than 0.001 a.u.

Synthetic Details

Methyl 5-methoxy-2-(4-methoxybenzyl)benzoate (**6**)[13]

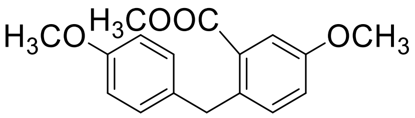

A 100-mL flask was charged with methyl 2-(bromomethyl)-5-methoxybenzoate (4.50 g, 17.4 mmol), (4-methoxyphenyl)boronic acid (4.00 g, 26.2 mmol), Cs_2CO_3 (22.8 g, 69.8 mmol), 1,2-dimethoxyethane (15 mL), and water (15 mL). After the reaction mixture was degassed by argon bubbling for 10 min, tetrakis(triphenylphosphino)palladium(0) ($Pd(PPh_3)_4$) (802 mg, 0.703 mmol) was added under argon atmosphere. The reaction mixture was refluxed overnight under an argon atmosphere. The resulting mixture was cooled to room temperature and extracted three times with EtOAc (25 mL). The separated organic phases were combined, washed with brine, dried over $MgSO_4$, and evaporated. The residue was purified by silica gel column chromatography to give the title compound (3.50 g, 74% yield) as colorless oil. All spectral data were in agreement with the literature.[13] ^{1}H NMR (300 MHz, DMSO-d_6) δ 7.25 (d, J = 2.6 Hz, 1H), 7.22 (d, J = 8.8 Hz, 1H), 7.08 (dd, J = 8.6, 2.8 Hz, 1H), 7.01 (d, J = 8.2 Hz, 2H), 6.80 (d, J = 8.2 Hz, 2H), 4.11 (s, 2H), 3.79 (s, 6H), 3.75 (s, 3H). ^{13}C NMR (75 MHz, DMSO-d_6) δ 167.44, 157.33, 157.21, 133.86, 133.15, 132.54, 130.54, 129.40, 117.88, 114.68, 113.61, 55.26, 36.82.

2,7-Dimethoxyanthracen-9(10*H*)-one (**7**)[14]

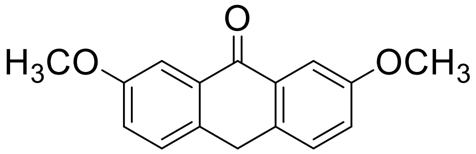

To a solution of compound **6** (3.50 g, 12.86 mmol) in 50 mL of CH_2Cl_2, trifluoromethanesulfonic acid (3.86 g, 25.7 mmol) was added dropwise at 0 °C under the argon atmosphere. The resulting mixture was allowed to warm to room temperature and stirred for 3 h. The reaction mixture was poured into water (500 mL) and extracted three times with EtOAc (40 mL). The separated organic phases were combined, washed with brine, dried over $MgSO_4$, and evaporated. The residue was purified by silica gel column chromatography to give the title compound (2.50 g, 77% yield) as light yellow solid. All spectral data were in agreement with the literature.[14] ^{1}H NMR (300 MHz, $CDCl_3$) δ 7.85 (d, J = 2.9 Hz, 2H), 7.41 (d, J = 8.5 Hz, 2H), 7.22 (dd, J = 8.6, 2.9 Hz, 2H), 4.26 (s, 2H), 3.95 (s, 6H). ^{13}C NMR (75 MHz, $CDCl_3$) δ 184.05, 158.64, 133.49, 132.70, 129.78, 121.79, 108.86, 55.60, 31.16.

2,7-Dimethoxy-9,9'-bianthryl (**8**)

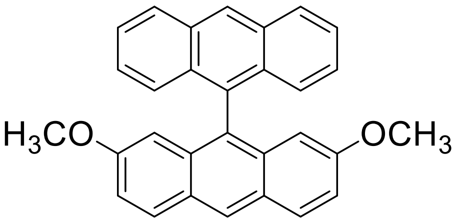

To a solution of 9-bromoanthracene (547 mg, 2.16 mmol) in anhydrous diethyl ether (100 mL), *n*-butyllithium (*n*-BuLi) (1.6 M in hexane, 1.9 mL, 3.1 mmol) was added dropwise at 0 °C under argon atmosphere. After reaction mixture was stirred for 10 min, compound **7** (500 mg, 1.96 mmol) was added. Then, the resulting mixture was stirred overnight at room temperature. The reaction mixture was quenched with methanol (10 mL) and evaporated. The residue was dissolved in toluene (30 mL), and refluxed with a catalytic amount of *p*-toluenesulfonic acid (about 3 mg) for 30 min. The resulting mixture was poured into 100 mL of water and extracted with CH_2Cl_2 (20 mL) for three times. The organic layers were combined, washed with brine, dried over $MgSO_4$, and evaporated. The residue was purified by silica gel chromatography to give title compound (160 mg, 20 % yield) as light yellow solid. ^{1}H NMR (300 MHz, CD_2Cl_2) δ 8.72 (s, 1H), 8.58 (s, 1H), 8.20 (d, J = 8.5 Hz, 2H), 8.07 (d, J = 9.2 Hz, 2H), 7.54 – 7.43 (m, 2H), 7.23 – 7.07 (m, 6H), 6.17 (t, J = 4.8 Hz, 2H), 3.28 (s, 6H). ^{13}C NMR (75 MHz, CD_2Cl_2) δ 158.10, 133.47, 132.19, 131.55, 130.74, 128.97, 127.53, 126.91, 126.08, 125.63, 119.19, 102.98, 30.07. HRMS (MALDI-TOF): *m/z* Calcd. For $C_{30}H_{22}O_2$: 414.1620, $[M]^+$, found: 414.1644.

9,9'-Bianthryl 2,7-bis(trifluoromethanesulfonate) (**9**)

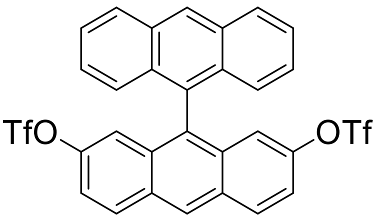

A 100-mL Schlenk tube was charged with compound **8** (140 mg, 0.340 mmol) and dry CH_2Cl_2(30 mL). Then, BBr_3 (1.0 M in dichloromethane, 6.8 mL, 6.8 mmol) was added dropwise to the solution at 0 °C. The resulting mixture was allowed to warm to room temperature and stirred for 12 h. The reaction mixture was poured into water (100 mL) and extracted three times with CH_2Cl_2 (20 mL). The organic phases were combined, washed with brine, dried over $MgSO_4$, and evaporated. The obtained 2,7-dihydroxy-9,9'-bianthryl was used directly for the next step without further purification. The crude product was dissolved in anhydrous CH_2Cl_2 (20 mL) and cooled to 0 °C. Then, pyridine (0.220 mL, 2.70 mmol) was added dropwise at 0 °C. After stirring for 10 min, trifluoromethanesulfonic anhydride (Tf_2O) (1.0 M in CH_2Cl_2, 1.0 mL, 1.0 mmol) was added dropwise under argon atmosphere at 0 °C. The resulting mixture was allowed to warm to room temperature and stirred for 12 h. The reaction mixture was poured

into 50 mL of water and extracted with CH_2Cl_2 (20 mL) for three times. The organic layers were combined, washed with brine, dried over $MgSO_4$, and evaporated. The residue was purified by silica gel column chromatography to give the title compound (130 mg, 58% yield) as light yellow solid. ^{1}H NMR (300 MHz, CD_2Cl_2) δ 8.78 (s, 1H), 8.72 (s, 1H), 8.25 (d, J = 9.3 Hz, 2H), 8.14 (d, J = 8.5 Hz, 2H), 7.47 – 7.31 (m, 4H), 7.13 (t, J = 7.7 Hz, 2H), 6.88 (dd, J = 6.0, 3.2 Hz, 4H). ^{13}C NMR (75 MHz, CD_2Cl_2) δ 148.24, 135.93, 132.43, 132.21, 131.89, 131.74, 131.10, 129.84, 129.20, 129.10, 128.61, 126.89, 125.97, 125.84, 120.94, 118.44, 116.72. HRMS (MALDI-TOF): m/z Calcd. For $C_{30}H_{16}F_6O_6S_2$: 650.0292, $[M]^+$, found: 650.0238.

2,7-Dibromo-9,9'-bianthryl (**3**)

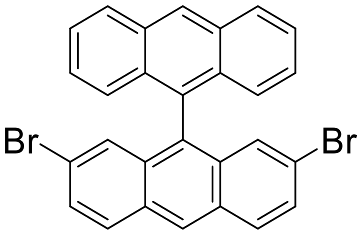

A 50-mL Schlenk tube was charged with $Pd_2(dba)_3$ (16.0 mg, 18.0 μmol), *t*-BuBrettPhos (27.0 mg, 28.0 μmol), and anhydrous 1,4-dioxane (4.0 mL) under argon atmosphere. The resulting mixture was heated at 120 °C and stirred at this temperature for 30 min. The catalyst solution was cooled to room temperature and added into a dried Schlenk tube charged with compound **9** (100 mg, 0.150 mmol), KF (8.93 mg, 0.150 mmol), and KBr (366.5 mg, 3.08 mmol). The reaction mixture was stirred vigorously at 130 °C for 12 h. The reaction mixture was poured into water (30 mL) and extracted with CH_2Cl_2 (20 mL) for three times. The organic layers were combined, washed with brine, dried over $MgSO_4$, and evaporated. The residue was purified by silica gel column chromatography to give the title compound (41.0 mg, 52% yield) as light yellow solid. ^{1}H NMR (300 MHz, CD_2Cl_2) δ 8.80 (s, 1H), 8.71 (s, 1H), 8.25 (d, J = 8.6 Hz, 2H), 8.09 (d, J = 9.0 Hz, 2H), 7.64 – 7.48 (m, 4H), 7.32 – 7.16 (m, 4H), 7.03 (d, J = 8.8 Hz, 2H). ^{13}C NMR (75 MHz, CD_2Cl_2) δ 133.27, 132.31, 132.03, 131.86, 130.93, 130.37, 129.71, 129.20, 128.55, 128.52, 128.34, 126.67, 126.43, 125.83, 121.54. HRMS (MALDI-TOF): m/z Calcd. For $C_{28}H_{16}Br_2$: 509.9619, $[M]^+$, found: 509.9669.

Additional STM and nc-AFM measurements results

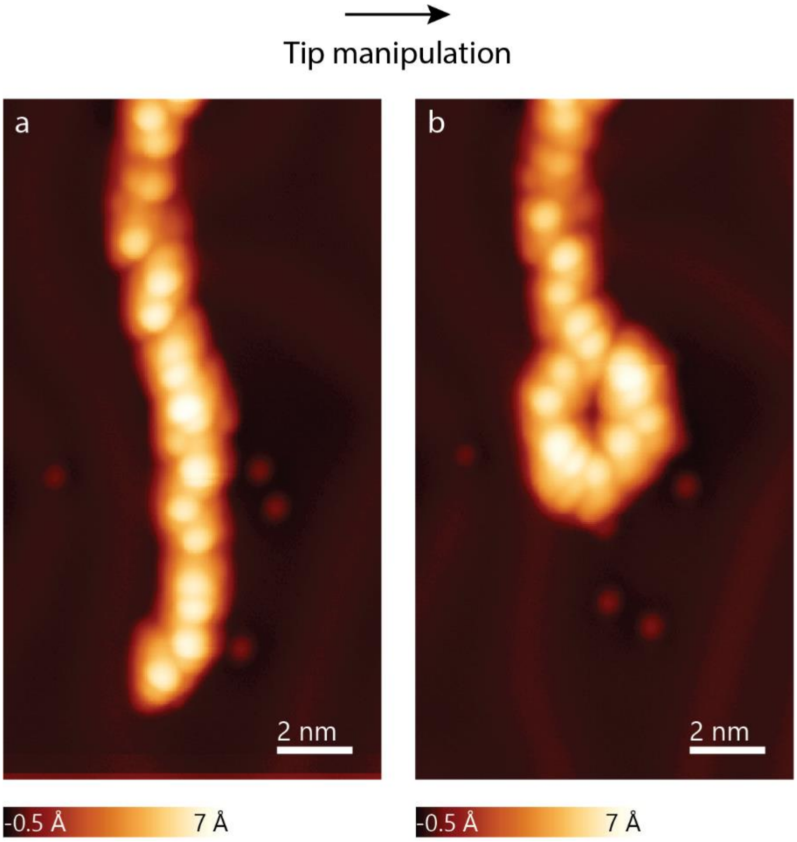

Figure S1. Constant-current STM images of polymer **10** before and after the tip manipulation are shown in a and b (V = -1.5 V, I = 50 pA, colorbar indicate the heights). After the image in a was acquired, the tip was approached closer to the surface with the tunneling parameters V = 10 mV and I = 1 nA. The tip was then made to move in a straight line upwards along the polymer. The STM image shown in b was acquired after the tip manipulation, revealing the intact but different adsorption geometry of the polymer, evidencing covalent linking between the individual monomers.

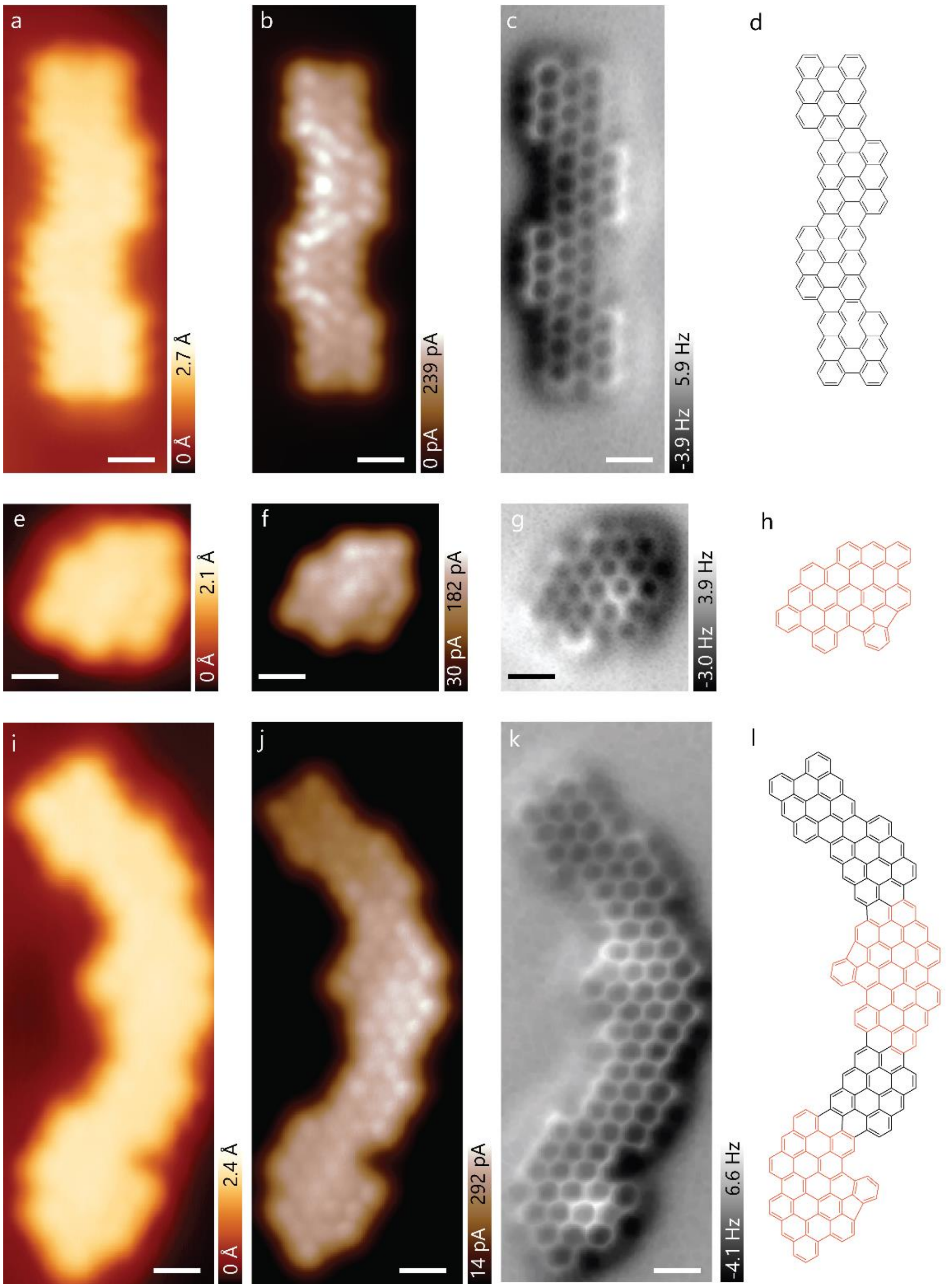

Figure S2. Constant-current STM images of different products after the cyclodehydrogenation step are shown in a, e and i. (V = 20 mV, I = 100pA, colorbars indicate height). Constant-height STM images are shown in b, f and j, these were acquired simultaneously with the nc-AFM images (Colorbars indicate current). Nc-AFM images are shown in c, g and k. The feedback loop was switched off (Δz = 0) on top of the GNR segments at 20 mV, 100 pA (colorbars indicate the frequency shift). The chemical structure of the GNR segment in a-c is shown in d, similarly, that of the segment in e-g is shown in h, and the segment in i-k in l. All scale bars are 0.5 nm.

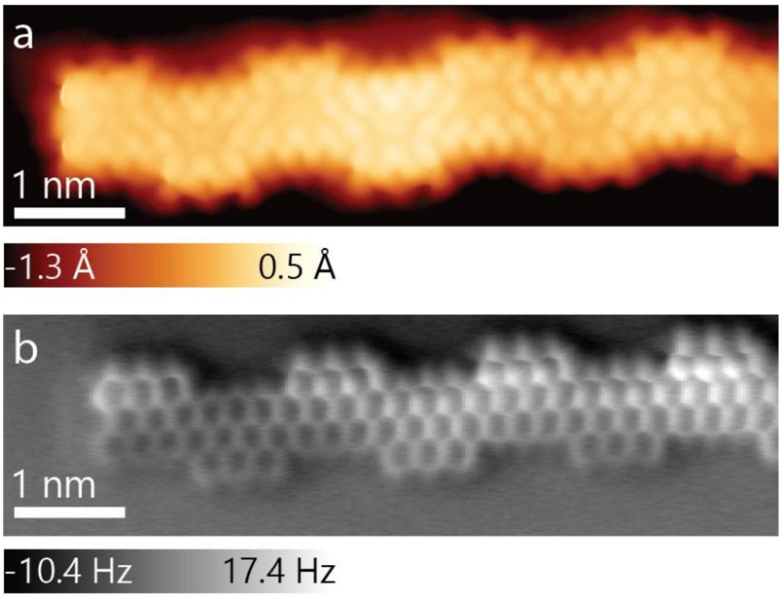

Figure S3. (a) STM and (b) nc-AFM characterization of a 7-unit-long 3-ZGNR-EA. The tunneling parameters for the STM are V = 10 mV, I = 190 pA (colorbars in a indicate height). The feedback loop was switched off (Δz = 0) on top of the GNR segments at V = 10 mV, I = 100 pA (colorbars in b indicate the frequency shift).

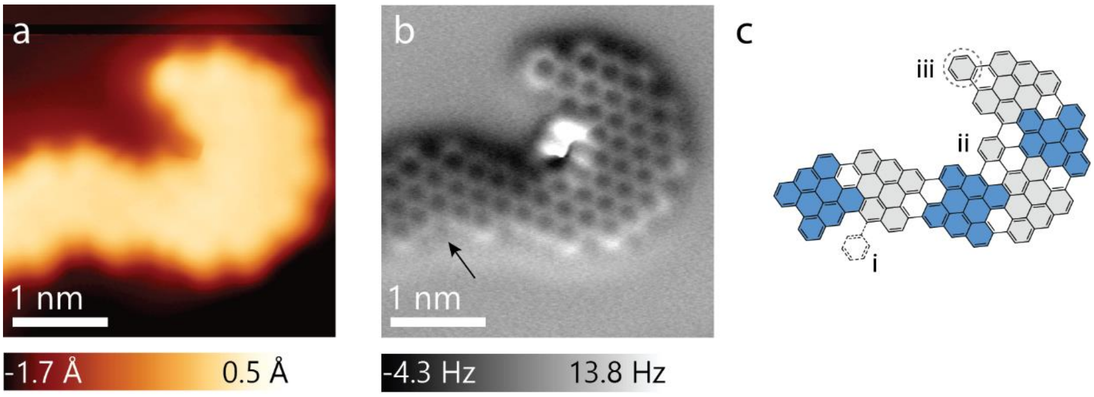

Figure S4. (a) STM and (b) nc-AFM images of a GNR segment. Panel c displays the chemical structure deduced from the nc-AFM image, with rings derived from a single precursor molecule distinctly colored to emphasize observed ring rearrangements. The initial finding, marked as 'i' in panel c, reveals the absence of a phenylene ring, also indicated by a black arrow in panel b, which can be explained by the extrusion of benzyne as discussed in the main text. Furthermore, a bright protrusion in the nc-AFM image is attributed to a cove-edge, which is twisted out of plane due to steric repulsion of C-H bonds (labelled 'ii'). Lastly, an additional ring, situated atop the segment can be seen (marked 'iii'), indicating ring migration. The tunneling parameters for the STM image are V = 20 mV, I = 100 pA (colorbars in a indicate height). The feedback loop was switched off (Δz = 0) on top of the GNR segments at V = 20 mV, I = 100 pA (colorbars in b indicate the frequency shift).

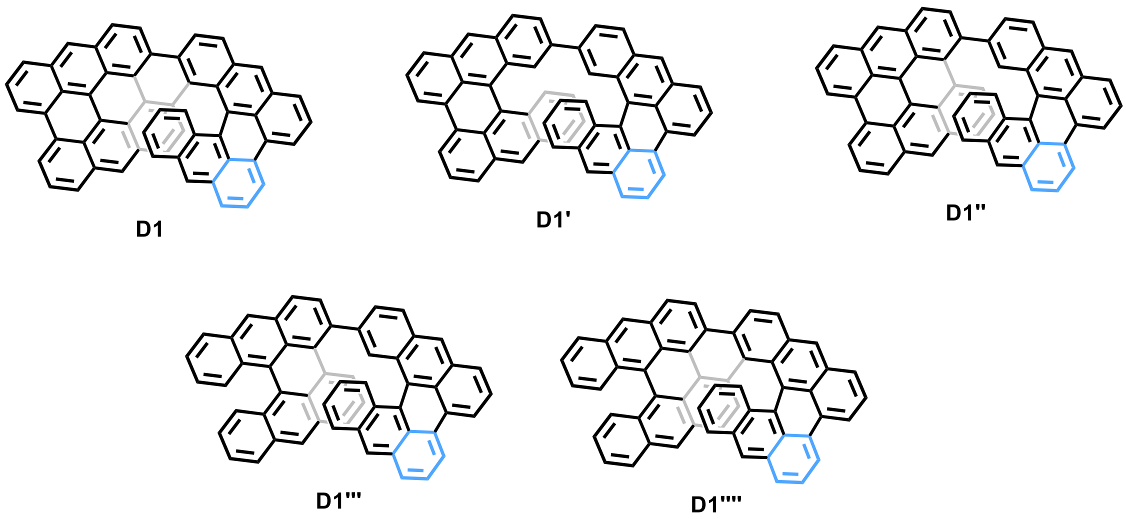

Figure S5. Possible intermediate structures after the partial cyclodehydrogenation of **11b**.

NMR Spectra

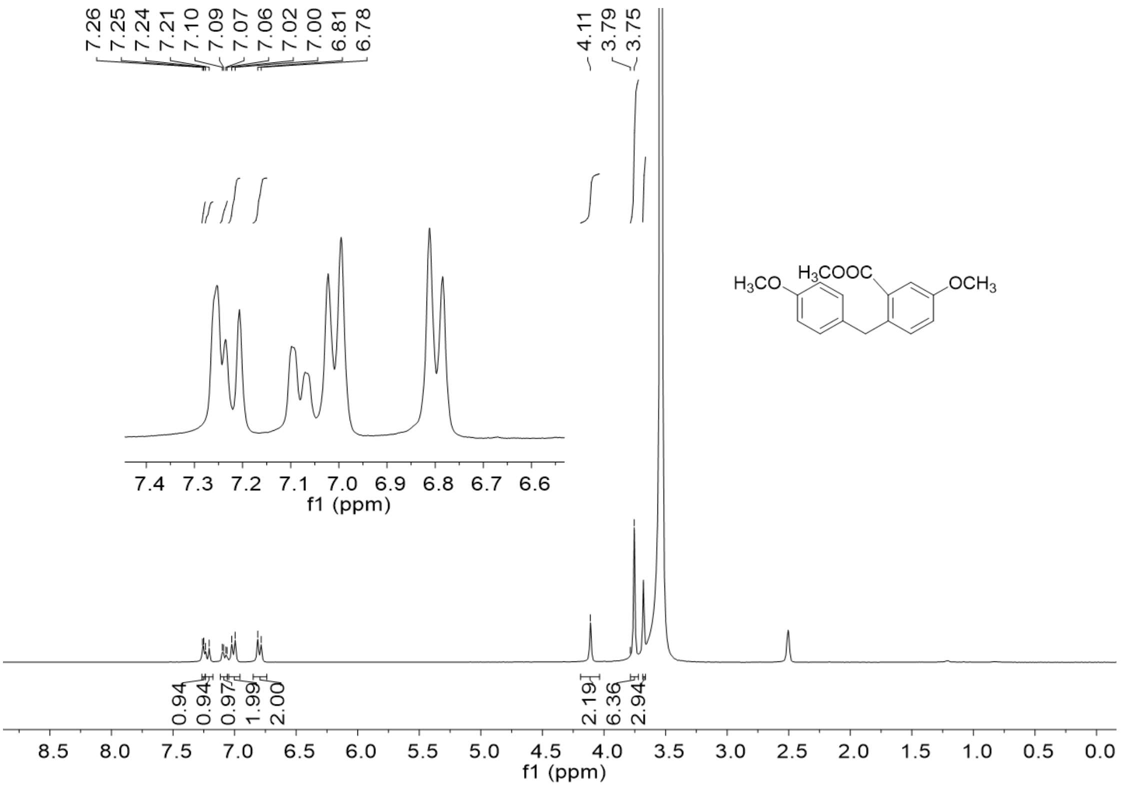

Figure S6. ^{1}H NMR spectrum of compound **6** in DMSO-d_6 (300 MHz, 298 K).

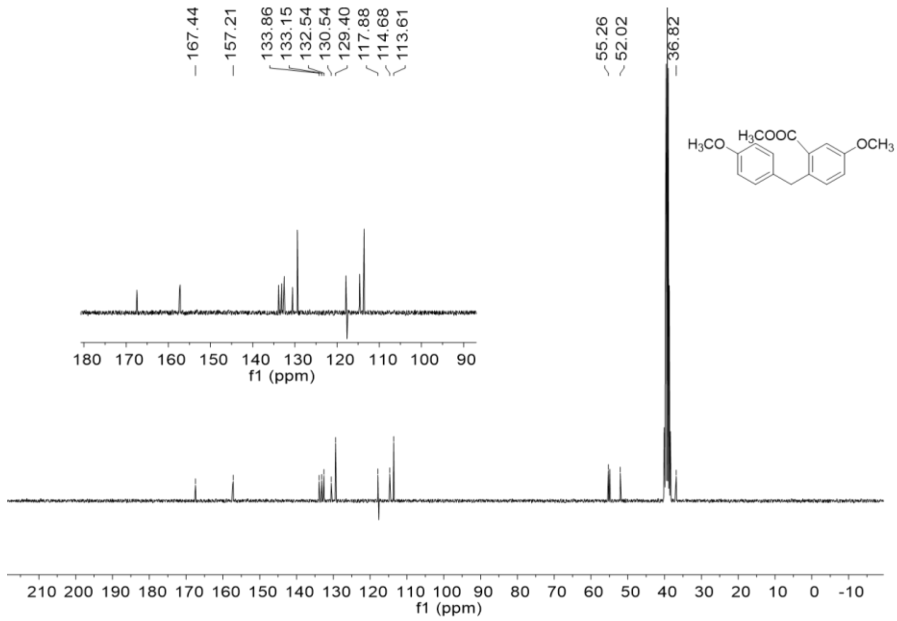

Figure S7. ^{13}C NMR spectrum of compound **6** in DMSO-d_6 (75 MHz, 298 K).

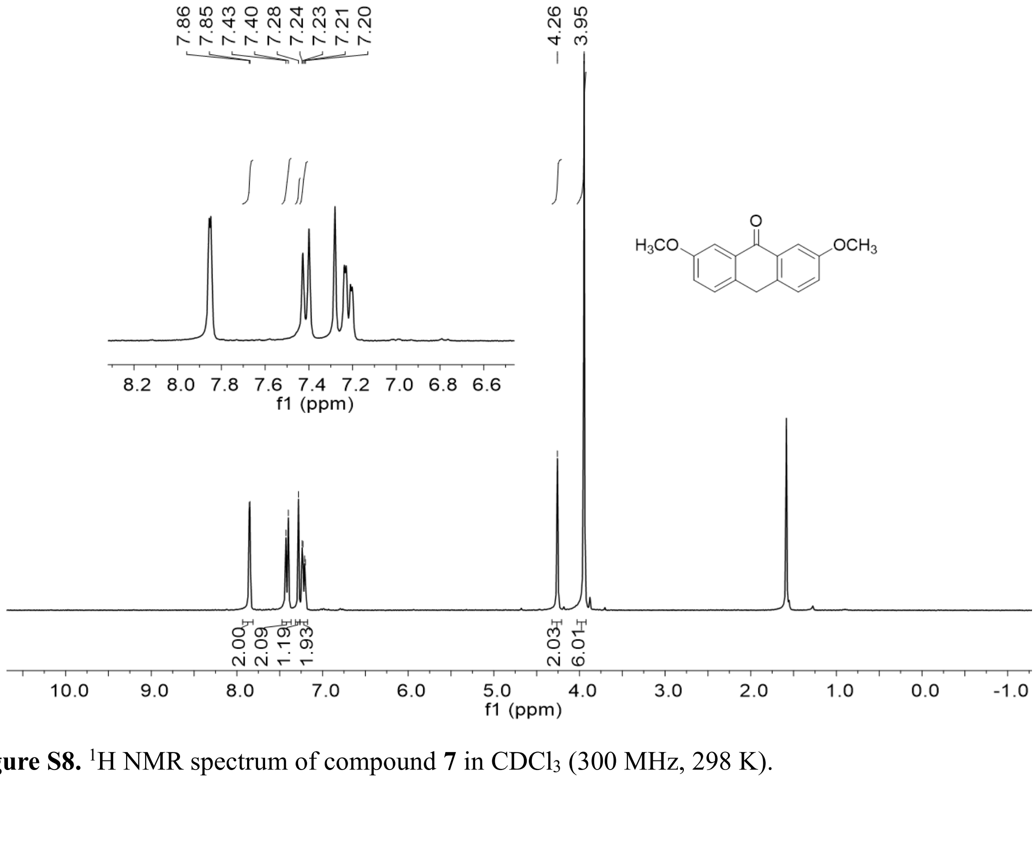

Figure S8. ^{1}H NMR spectrum of compound **7** in $CDCl_3$ (300 MHz, 298 K).

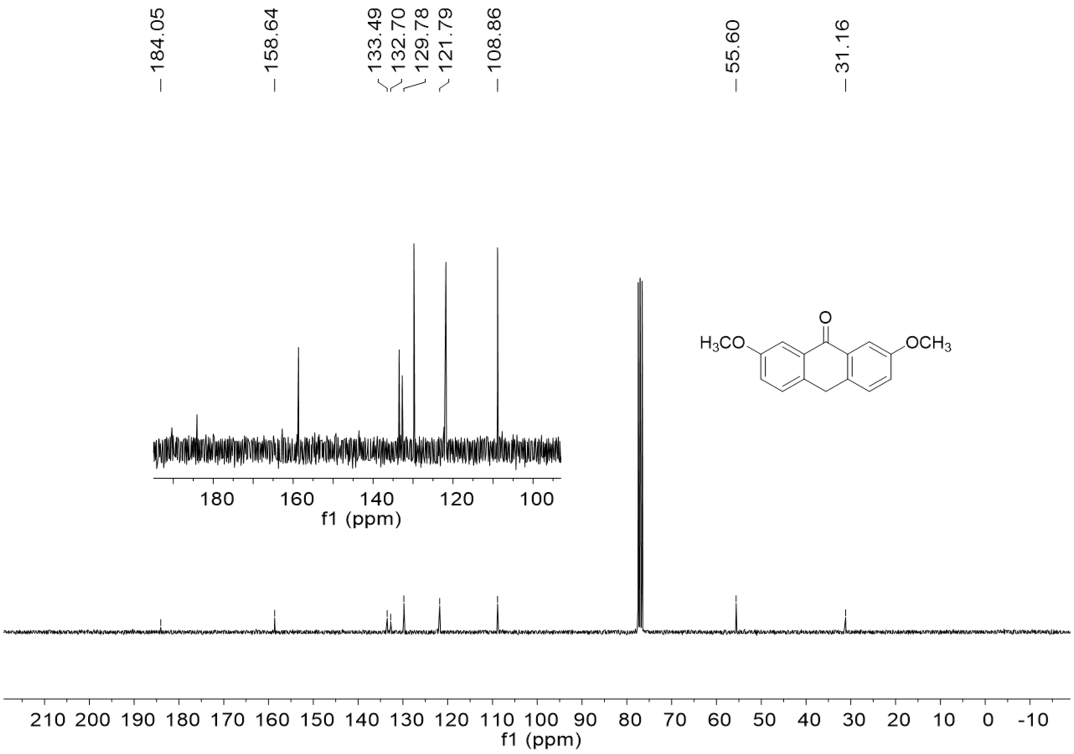

Figure S9. ^{13}C NMR spectrum of compound **7** in $CDCl_3$ (75 MHz, 298 K).

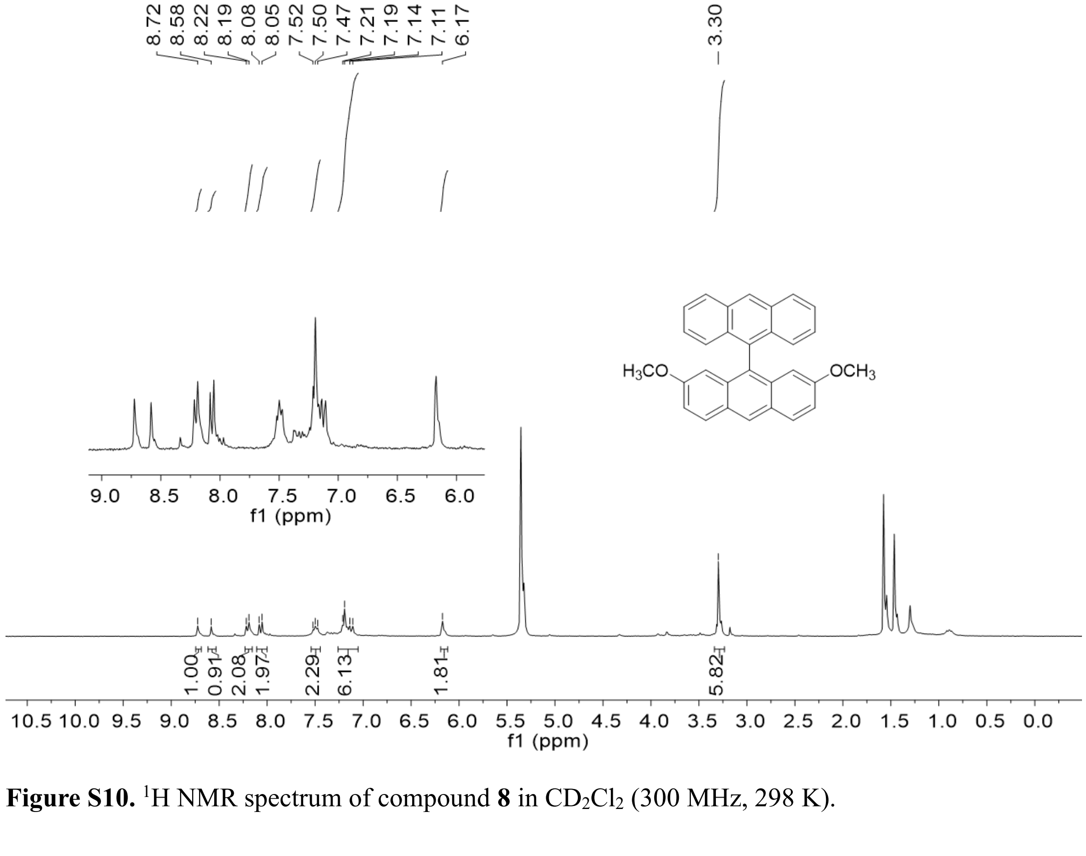

Figure S10. ^{1}H NMR spectrum of compound **8** in CD_2Cl_2 (300 MHz, 298 K).

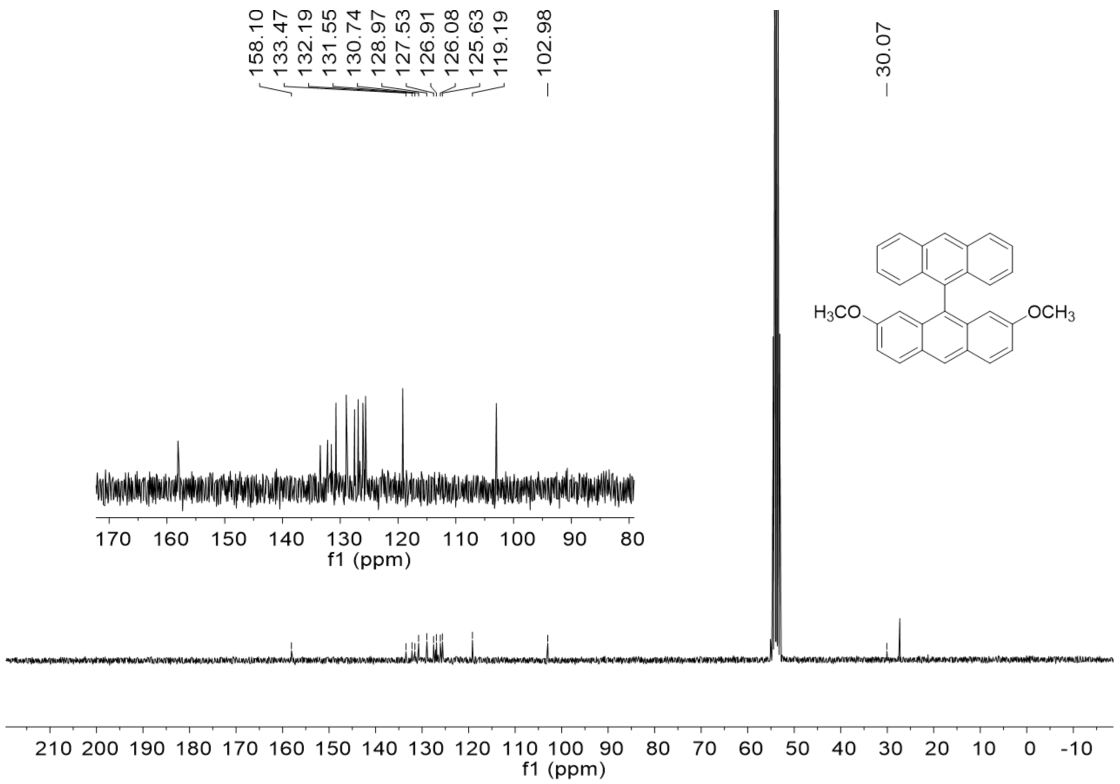

Figure S11. ^{13}C NMR spectrum of compound **8** in CD_2Cl_2 (75 MHz, 298 K).

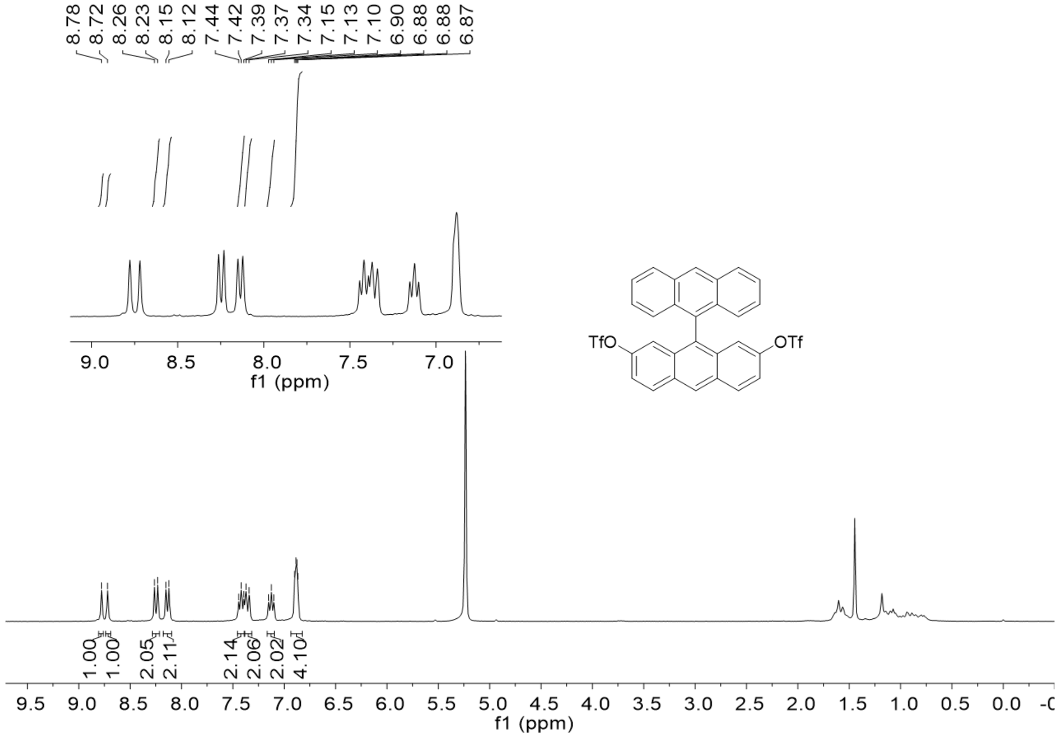

Figure S12. ^{1}H NMR spectrum of compound **9** in CD_2Cl_2 (300 MHz, 298 K).

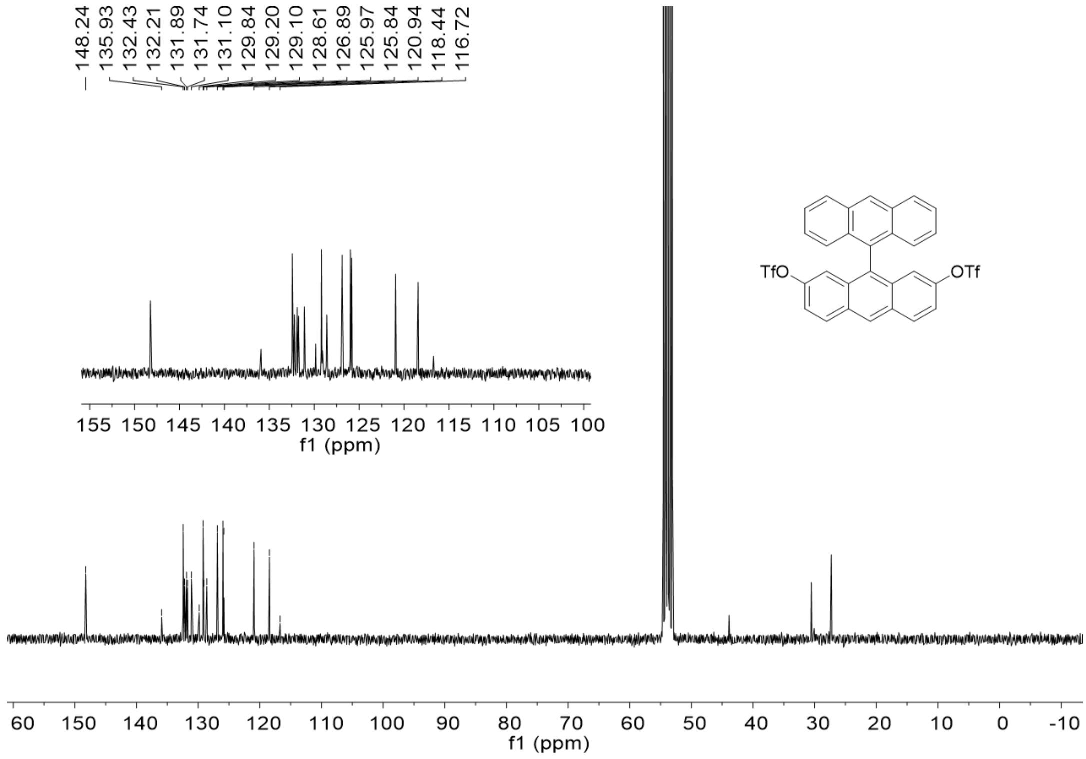

Figure S13. ^{13}C NMR spectrum of compound **9** in CD_2Cl_2 (75 MHz, 298 K).

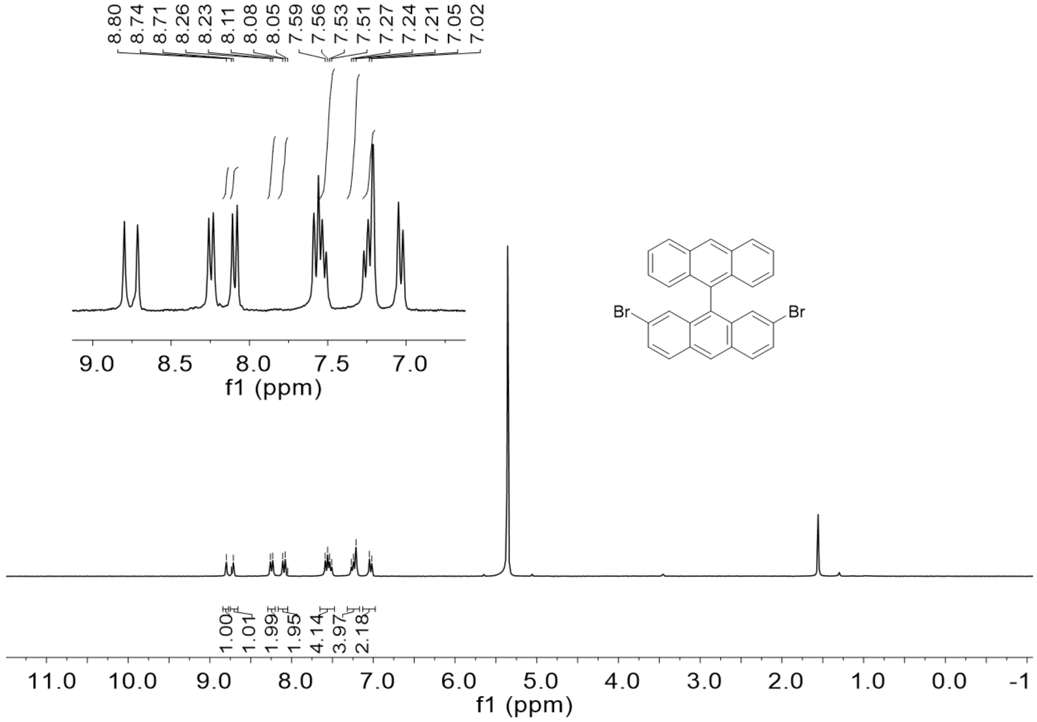

Figure S14. ^{1}H NMR spectrum of compound **3** in CD_2Cl_2 (300 MHz, 298 K).

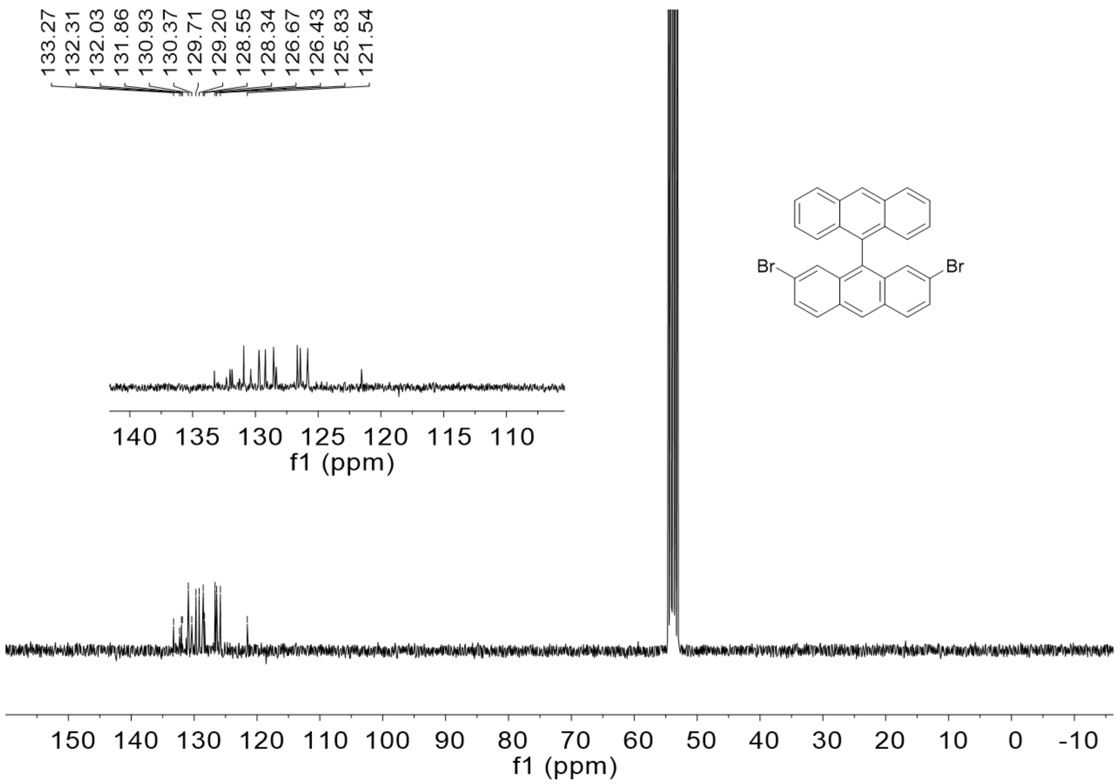

Figure S15. ^{13}C NMR spectrum of compound **3** in CD_2Cl_2 (75 MHz, 298 K).

References:

(1) Giessibl, F. J., Atomic resolution on Si(111)-(7×7) by noncontact atomic force microscopy with a force sensor based on a quartz tuning fork. *Appl. Phys. Lett.* **2000,** *76*, 1470-1472.

(2) Bartels, L.; Meyer, G.; Rieder, K. H.; Velic, D.; Knoesel, E.; Hotzel, A.; Wolf, M.; Ertl, G., Dynamics of Electron-Induced Manipulation of Individual CO Molecules on Cu(111). *Phys. Rev. Lett.* **1998,** *80*, 2004-2007.

(3) Yakutovich, A. V.; Eimre, K.; Schütt, O.; Talirz, L.; Adorf, C. S.; Andersen, C. W.; Ditler, E.; Du, D.; Passerone, D.; Smit, B.; Marzari, N.; Pizzi, G.; Pignedoli, C. A., AiiDAlab – an ecosystem for developing, executing, and sharing scientific workflows. *Comput. Mater. Sci.* **2021,** *188*, 110165.

(4) Pizzi, G.; Cepellotti, A.; Sabatini, R.; Marzari, N.; Kozinsky, B., AiiDA: automated interactive infrastructure and database for computational science. *Comput. Mater. Sci.* **2016,** *111*, 218-230.

(5) Hutter, J.; Iannuzzi, M.; Schiffmann, F.; VandeVondele, J., cp2k: atomistic simulations of condensed matter systems. *WIREs Computational Molecular Science* **2014,** *4*, 15-25.

(6) VandeVondele, J.; Hutter, J., Gaussian basis sets for accurate calculations on molecular systems in gas and condensed phases. *J. Chem. Phys.* **2007,** *127*, 114105.

(7) Goedecker, S.; Teter, M.; Hutter, J., Separable dual-space Gaussian pseudopotentials. *Phys. Rev. B* **1996,** *54*, 1703-1710.

(8) Perdew, J. P.; Burke, K.; Ernzerhof, M., Generalized Gradient Approximation Made Simple. *Phys. Rev. Lett.* **1996,** *77*, 3865-3868.

(9) Grimme, S.; Antony, J.; Ehrlich, S.; Krieg, H., A consistent and accurate ab initio parametrization of density functional dispersion correction (DFT-D) for the 94 elements H-Pu. *J. Chem. Phys.* **2010,** *132*, 154104.

(10) Hapala, P.; Kichin, G.; Wagner, C.; Tautz, F. S.; Temirov, R.; Jelínek, P., Mechanism of high-resolution STM/AFM imaging with functionalized tips. *Phys. Rev. B* **2014,** *90*, 085421.

(11) Giannozzi, P.; Baseggio, O.; Bonfà, P.; Brunato, D.; Car, R.; Carnimeo, I.; Cavazzoni, C.; de Gironcoli, S.; Delugas, P.; Ferrari Ruffino, F.; Ferretti, A.; Marzari, N.; Timrov, I.; Urru, A.; Baroni, S., Quantum ESPRESSO toward the exascale. *J. Chem. Phys.* **2020,** *152*, 154105.

(12) Lejaeghere, K.; Bihlmayer, G.; Björkman, T.; Blaha, P.; Blügel, S.; Blum, V.; Caliste, D.; Castelli, I. E.; Clark, S. J.; Dal Corso, A.; de Gironcoli, S.; Deutsch, T.; Dewhurst, J. K.; Di Marco, I.; Draxl, C.; Dułak, M.; Eriksson, O.; Flores-Livas, J. A.; Garrity, K. F.; Genovese, L.; Giannozzi, P.; Giantomassi, M.; Goedecker, S.; Gonze, X.; Grånäs, O.; Gross, E. K. U.; Gulans, A.; Gygi, F.; Hamann, D. R.; Hasnip, P. J.; Holzwarth, N. A. W.; Iuşan, D.; Jochym, D. B.; Jollet, F.; Jones, D.; Kresse, G.; Koepernik, K.; Küçükbenli, E.; Kvashnin, Y. O.; Locht, I. L. M.; Lubeck, S.; Marsman, M.; Marzari, N.; Nitzsche, U.; Nordström, L.; Ozaki, T.; Paulatto, L.; Pickard, C. J.; Poelmans, W.; Probert, M. I. J.; Refson, K.; Richter, M.; Rignanese, G.-M.; Saha, S.; Scheffler, M.; Schlipf, M.; Schwarz, K.; Sharma, S.; Tavazza, F.; Thunström, P.; Tkatchenko, A.; Torrent, M.; Vanderbilt, D.; van Setten, M. J.; Van Speybroeck, V.; Wills, J. M.; Yates, J. R.; Zhang, G.-X.; Cottenier, S., Reproducibility in density functional theory calculations of solids. *Science* **2016,** *351*, aad3000.

(13) Zhu, L.; Ren, X. J.; Yu, Y. H.; Ou, P. C.; Wang, Z. X.; Huang, X. L., Palladium-Catalyzed Three-Component Coupling Reaction of o-Bromobenzaldehyde, N-Tosylhydrazone, and Methanol. *Org. Lett.* **2020,** *22*, 2087-2092.

(14) Hirao, Y.; Hamamoto, Y.; Nagamachi, N.; Kubo, T., Solvent viscosity-dependent isomerization equilibrium of tetramethoxy-substituted bianthrone. *Phys. Chem. Chem. Phys.* **2019,** *21*, 12209-12216.
